# Exploring Meiotic Recombination and Its Potential Benefits in South African Beef Cattle: A Review

**DOI:** 10.3390/vetsci12070669

**Published:** 2025-07-16

**Authors:** Nozipho A. Magagula, Keabetswe T. Ncube, Avhashoni A. Zwane, Bohani Mtileni

**Affiliations:** 1 Department of Animal Sciences, Tshwane University of Technology, Pretoria 0001, South Africa; 2 Animal Breeding and Genetics, Agricultural Research Council-Animal Production, Irene 0062, South Africa; 3 Department of Biochemistry, Genetics and Microbiology, University of Pretoria, Hatfield, Pretoria 0002, South Africa

**Keywords:** evolution, meiosis, molecular genetics, sexual reproduction, recombination

## Abstract

Meiotic recombination is a key evolutionary process that generates novel allele combinations during prophase I of meiosis, promoting genetic diversity and enabling the selection of desirable traits in livestock breeding. Although its molecular mechanisms are well-characterised in model organisms such as humans and mice, studies in African indigenous cattle, particularly South African breeds, remain scarce. Key regulators of recombination, including *PRDM9*, *SPO11*, and *DMC1*, play essential roles in crossover formation and genome stability, with mutations in these genes often linked to fertility defects. Despite the Bonsmara and Nguni breeds’ exceptional adaptability to arid and resource-limited environments, little is known about how recombination contributes to their unique genetic architecture and adaptive traits. This review synthesises the current knowledge on the molecular basis of meiotic recombination, with a focus on prophase I events and associated structural proteins and enzymes. It also highlights the utility of genome-wide tools, particularly high-density single nucleotide polymorphism (SNP) markers for recombination mapping. By focusing on the underexplored recombination landscape in South African beef cattle, this review identifies key knowledge gaps. It outlines how recombination studies can inform breeding strategies aimed at enhancing genetic improvement, conservation, and the long-term sustainability of local beef production systems.

## Simple Summary

By generating novel allele combinations during prophase I of meiosis, meiotic recombination is a fundamental evolutionary mechanism that enhances genetic diversity and supports the selection of desirable traits in livestock breeding. This process is crucial for improving the genetic potential of livestock through selective breeding. While recombination has been studied in commercial cattle breeds from Europe and North America, it remains unexplored and not understood in South African beef cattle, especially the Bonsmara and Nguni breeds. These indigenous breeds are highly valued for their adaptability to harsh, resource-limited environments, yet the genetic mechanisms underlying their resilience and productivity are not well characterised. This review explores how genome-wide technologies, especially those using high-density single nucleotide polymorphism (SNP) markers, can be used to map recombination patterns and identify genes involved in key traits. Applying these tools to South African cattle could enhance breeding strategies by improving the accuracy of selecting animals with a superior genetic merit. Understanding recombination in these breeds will also support their conservation and sustainable use. Ultimately, this knowledge has significant implications for advancing food security, promoting rural development, and ensuring the long-term adaptability of South African beef cattle under climate change.

## 1. Introduction

Meiotic recombination is a fundamental biological mechanism in sexually reproducing organisms that enables the exchange of genetic material between homologous chromosomes during prophase I of meiosis. This process results in the formation of crossover events that generate novel allele combinations in gametes, thereby increasing the genetic variation within populations. In livestock species, including cattle, meiotic recombination is a crucial evolutionary force that enhances genetic diversity and facilitates the selection and propagation of economically important traits, such as fertility, carcass composition, the growth rate, feed efficiency, and resistance to infectious diseases [1,2]. As such, recombination plays an integral role in both natural adaptation and artificial selection within breeding programmes.

Recombination events are not randomly distributed throughout the genome but tend to occur in specific, narrow regions termed recombination hotspots, which are typically 1–2 kb in length [3,4]. In many mammals, the location of these hotspots is determined by the binding of the *PRDM9* protein. This meiosis-specific histone methyltransferase recognises specific DNA motifs through its zinc finger domain [5,6]. The identification of recombination hotspots and the elucidation of the molecular machinery governing crossover formation have significantly improved our understanding of genome evolution and the inheritance of complex traits in model species such as humans and mice. However, the recombination landscape of livestock species, particularly African indigenous and composite breeds, remains poorly characterised.

In cattle, meiotic recombination not only contributes to genomic variation but also shapes linkage disequilibrium patterns, haplotype structures, and the inheritance of quantitative trait loci (QTLs). As such, it influences the resolution and power of genome-wide association studies (GWASs) and affects the accuracy of genomic estimated breeding values (GEBVs) in genomic selection. A deeper understanding of the rates, distribution, and determinants of recombination, especially their variation across sexes and breeds, can improve marker-assisted selection and the optimisation of breeding schemes.

This review focuses specifically on the potential benefits of studying meiotic recombination in South African beef cattle, using Bonsmara and Nguni breeds as exemplars. The Bonsmara is a scientifically bred composite breed developed through the structured crossbreeding of Afrikaner, Hereford, and Shorthorn cattle, optimised for beef production under local conditions. The Nguni, by contrast, is an indigenous Sanga-type breed with a long history of adaptation to extensive pastoralism, renowned for its disease resistance, tick tolerance, thermotolerance, and reproductive efficiency [7,8]. These breeds represent valuable genetic resources for sustainable livestock production under sub-Saharan African conditions. However, there remains limited knowledge about how recombination contributes to the maintenance and inheritance of their adaptive and productive traits.

Despite the increasing interest in the genetics of adaptation and climate resilience, the recombination landscape of South African beef cattle has not been comprehensively mapped. Key questions remain regarding whether recombination hotspots are conserved across breeds; whether recombination rates vary between sexes; and how genes such as *PRDM9*, *SPO11*, and *DMC1* modulate the frequency and distribution of crossover events in these populations. Furthermore, the limited application of high-density SNP genotyping and other high-throughput genomic tools in these breeds has hindered efforts to integrate recombination data into national genetic evaluation systems.

This review addresses these critical gaps by (i) describing the molecular events and structural proteins involved in meiotic recombination, with an emphasis on the crossover formation during prophase I; (ii) profiling key recombination-related genes, particularly *PRDM9*, *SPO11*, and *DMC1*, that regulate the initiation and resolution of recombination; and (iii) summarising the genomic tools and methodological approaches, especially those based on SNP markers used to study recombination in mammals. While these mechanisms have been elucidated in model species, their relevance and application in African cattle genetics remain underexplored. By contextualising recombination biology within the unique production systems of South African beef cattle, this review aims to lay a conceptual and technical foundation for future studies that leverage recombination to enhance the genetic gain, the breed resilience, and the sustainability of livestock farming in the region.

## 2. The Origin and Distribution of Bonsmara and Nguni Cattle in South Africa

Understanding the genomic architecture of South African beef cattle is fundamental to elucidating how meiotic recombination influences the genetic diversity, trait inheritance, and selection potential. Two of the most prominent beef breeds in South Africa, Bonsmara and Nguni, present distinct yet complementary genetic profiles that make them ideal candidates for studying recombination dynamics. This section provides an overview of their origin, phenotypic characteristics, population trends, and genomic features, establishing a biological and agricultural rationale for their inclusion in recombination research aimed at improving genetic gain and sustainability in beef production.

### 2.1. The Bonsmara

The Bonsmara is a composite synthetic breed developed through structured cross-matings and back-crosses involving 5/8 Afrikaner, 3/16 Shorthorn, and 3/16 Hereford cattle. The original breeding goal was to create a breed that merges the adaptability of indigenous cattle with the enhanced production potential of exotic Bos taurus breeds, particularly under South Africa’s subtropical and highveld environments [9,10,11,12]. Today, the Bonsmara is globally recognised as one of the few *Bos taurus indicus* composite breeds capable of thriving in tropical and subtropical regions [11].

**Figure 1:**
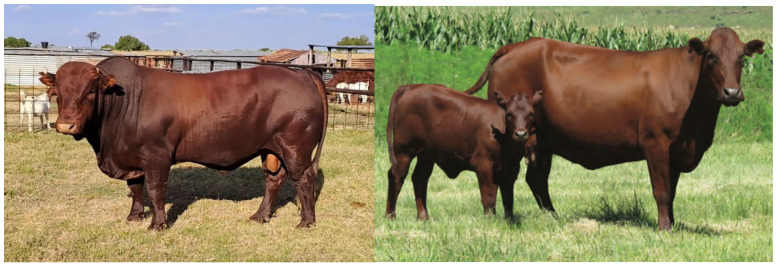
A Bonsmara bull (**left**) and a Bonsmara cow with a calf (**right**). (https://www.thecattlesite.com/breeds/beef/70/bonsmara/, accessed on: 16 February 2024) and (https://Bonsmara.co.za/journal/, accessed on: 16 February 2024).

Phenotypically, Bonsmara cattle exhibit a uniform red to brown coat, medium horns, and a slight cervicothoracic hump in bulls. They are known for their good beef conformation, with adult bulls averaging 950 kg and cows averaging about 618 kg. The average height at the withers in adult males is approximately 123 cm (Figure 1). The breed is further characterised by a superior meat quality, high weaning weights, early sexual maturity (12–15 months), and strong maternal traits (https://dad.fao.org.za/, accessed on: 16 February 2024). Notably, Bonsmara cattle show a significant tolerance to ticks and tick-borne diseases [13], enhancing their resilience in hot and humid conditions and making them particularly well-suited for low-input beef production systems.

Despite being one of South Africa’s most economically important beef breeds [9,14], the Bonsmara population has experienced fluctuations. In recent years, however, the population has shown growth, with a population of 142,849 reported in 2023, a 57,843 increase from 2018. This upward trend signifies the growing industry preference for the breed due to its commercial viability and suitability for both local and export markets (Table 1).

From a genomic standpoint, the Bonsmara has been extensively characterised using single nucleotide polymorphism (SNP) data. Makina et al. [8] reported moderate heterozygosity levels, consistent with its hybrid origin. Further, studies by Saranana et al. [15] validated the use of SNP panels to assess the genetic diversity and population structure across South African cattle breeds, including Bonsmara. These genomic insights provide a robust foundation for mapping recombination hotspots and investigating the role of recombination in trait development, particularly in fertility, tick resistance, and maternal efficiency.

Given its well-documented genomic resources, favourable production traits, and adaptive capacity, the Bonsmara serves as a model breed for elucidating the influence of meiotic recombination on genetic gain. The fine-scale mapping of recombination events in this breed could accelerate genomic selection and enable more precise breeding strategies to optimise productivity and resilience in commercial beef systems.

### 2.2. The Nguni

The Nguni is a hardy, indigenous Sanga-type breed, resulting from an ancient admixture between Bos indicus and Bos taurus cattle [16]. The name derives from the Nguni-speaking peoples of Southern Africa [17]. Nguni cattle are believed to have entered the region approximately 2000 years ago, brought by migratory agro-pastoralists moving from North and Central Africa [17]. Following their entry through the Limpopo River basin, tribal migration facilitated the development of geographically distinct ecotypes, such as Makhathini, Pedi, Shangaan, and Venda [17]. Today, Nguni cattle are predominant in both humid and arid zones of South Africa and are found in commercial as well as smallholder farming systems [18].

**Figure 2:**
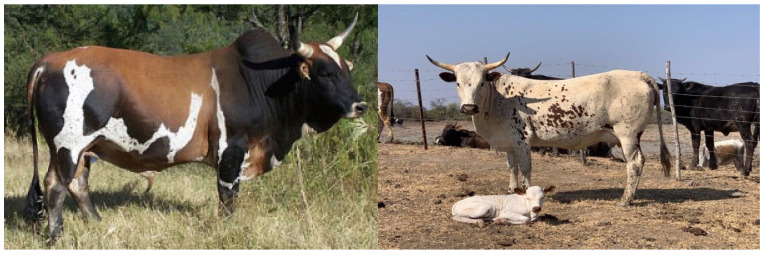
Nguni bull (**left**) and cow with calf (**right**) (https://www.thecattlesite.com/breeds/beef/93/nguni, accessed on: 16 February 2024).

Nguni cattle are best known for their polychromatic coat patterns, which are often black, white, brown, red, or cream in various combinations (Figure 2). Cows typically weigh 300–400 kg, while bulls range from 500 to 700 kg (https://thecattlesite.com, accessed on: 16 February 2024). Despite their smaller size compared to other beef breeds, Nguni cattle are exceptionally resilient and are well adapted to both arid and humid environments [17]. They exhibit a high tolerance to tick infestations, facilitated by their dense, glossy coats and fine hair, which inhibit tick attachment [13,19]. Moreover, their robust skeletal structure supports grazing over long distances and rugged terrain [7].

Nguni cows also possess a high fertility, low calf mortality at first calving, and stable reproductive performance, all of which enhance their utility in smallholder and communal farming systems [16].

From a genomic perspective, the Nguni breed is characterised by high genetic diversity and low inbreeding coefficients, reflecting its long evolutionary history and limited artificial selection pressure [8,15]. These attributes render Nguni cattle a valuable resource for studying how meiotic recombination contributes to the generation of adaptive traits. Given the likely polygenic basis of characteristics such as parasite resistance, heat tolerance, and fertility, it is plausible that recombination plays a significant role in the trait variation and persistence across generations.

However, fine-scale recombination patterns, including the identification of recombination hotspots and gene–environment interactions, remain largely unexplored in the Nguni breed. Unravelling these patterns can not only inform conservation strategies aimed at preserving indigenous genetic resources but also enable a genomic-assisted selection tailored to improving productivity in resource-constrained systems.

As shown in Table 2, Bonsmara and Nguni cattle represent contrasting genetic and phenotypic models for exploring meiotic recombination in South African beef production systems.

The Bonsmara cattle offer a structured genomic background and commercial performance advantages, making them ideal for trait mapping and marker-assisted selection. In contrast, Nguni cattle possess rich indigenous genetic diversity and resilience traits shaped by natural selection, offering a valuable context for understanding how recombination contributes to adaptive variation. These differences justify breed-specific investigations into recombination landscapes to optimise genomic selection strategies tailored to the distinct needs of each production system.

## 3. Meiotic Recombination Mechanism

Meiotic recombination is a pivotal biological process required by all sexually reproducing organisms, including cattle. It occurs during prophase I of meiosis, where diploid germ cells undergo a reductional division to form haploid gametes through the segregation of homologous chromosomes [20]. This exchange of genetic material introduces novel allele combinations, which underpin genetic diversity in offspring and play a fundamental role in trait inheritance, adaptive evolution, and genetic improvement in livestock breeding programmes.

**Figure 3:**
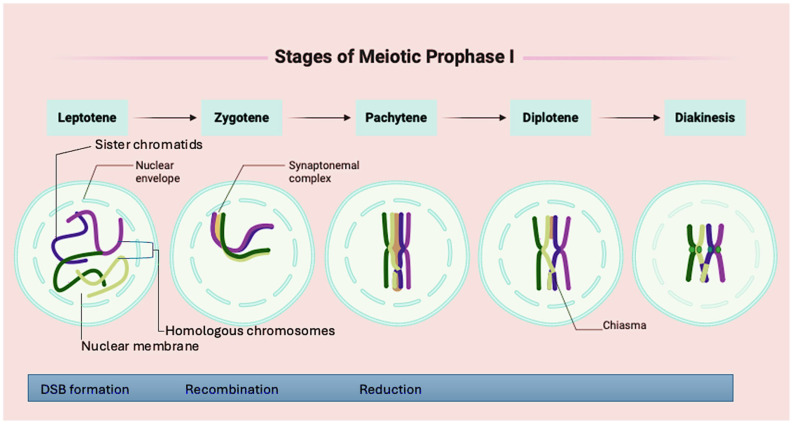
Stages of chromosomal organisation in prophase I of meiosis.

Prophase I is subdivided into five cytological stages, leptonema, zygonema, pachynema, diplonema, and diakinesis, each marked by distinct structural and molecular events that initiate, regulate, and complete recombination [21] (Figure 3). During the leptonema stage, chromosomes begin to condense into thin threads and attach to the nuclear envelope, forming chromatin loops from axial elements [22,23]. At this point, structural proteins such as REC8 and HORMAD1 start organising the chromosomal axes in preparation for homologous pairing. As meiosis progresses to the zygonema stage, homologous chromosomes initiate pairing and alignment. A programmed double-strand break (DSB), induced by the enzyme *SPO11*, marks the beginning of meiotic recombination [24]. Concurrently, a scaffold known as the synaptonemal complex (SC) begins to form between aligned chromosomes. This proteinaceous structure, composed of proteins such as *SYCP1*, *SYCP2*, and *SYCP3*, facilitates synapsis between homologs (Figure 3). The DSBs are processed by strand exchange proteins *RAD51* and *DMC1*, which facilitate strand invasion and homologue recognition.

In the pachynema stage, the SC fully develops along the paired homologs. Here, the repair of DSBs occurs via interhomolog recombination, leading to the formation of crossovers (COs). These COs result in the physical exchange of chromosomal regions between homologous chromosomes. Proteins such as *MLH1* and *MLH3* stabilise the recombination intermediates and ensure that only a subset of DSBs are resolved as COs. In the subsequent diplonema stage, the SC begins to disassemble unevenly, causing the desynapsis of the homologous chromosomes. As the chromosomes begin to separate, recombination events become cytologically visible in the form of chiasmata [22]. Finally, during the diakinesis stage, chromosomes continue to condense, and homologs remain joined at chiasmata and centromeres, marking the completion of prophase I and the transition to metaphase I [24].

Understanding the molecular mechanisms of meiotic recombination is not merely of academic interest; it has direct implications for beef cattle breeding and genomic selection. Recombination governs the reshuffling of alleles that determine complex traits such as fertility, disease resistance, feed efficiency, and carcass quality traits that are central to the productivity of South African beef breeds like Nguni and Bonsmara. Characterising where and how recombination occurs allows breeders and geneticists to map crossover hotspots, detect recombination-associated quantitative trait loci (QTLs), and identify genomic regions responsible for adaptation and resilience.

Moreover, the variation in the activity of key recombination-related genes, such as *PRDM9*, *DMC1*, *RAD51C*, and *XRCC3*, may underlie differences in the recombination rate and crossover distribution across cattle breeds. This variation can influence the efficacy of genomic selection and the resolution of genome-wide association studies (GWASs). In genetically diverse or admixed populations, such as those in South Africa, understanding recombination patterns is essential for refining marker-assisted selection, increasing the accuracy of genomic estimated breeding values (GEBVs), and preserving functional diversity in the breeding stock.

Therefore, linking the molecular details of prophase I to applied cattle breeding is crucial. Meiotic recombination not only creates new haplotype combinations for selection but also enhances the potential for long-term genetic gain and sustainable beef production.

## 4. Methods Used for the Detection of Recombination

Meiotic recombination can be studied using a range of approaches depending on the available genomic resources, the resolution required, the level of population structure, and the intended application. In livestock, particularly in genetically diverse and often admixed populations such as African indigenous cattle, the choice of the method also depends on the availability of pedigree records and the feasibility of large-scale sequencing. This section outlines three primary methods used to detect recombination events in mammals: pedigree-based, linkage disequilibrium (LD)-based, and gamete-based methods, highlighting their strengths, limitations, and relevance to cattle genomics.

### 4.1. Pedigree-Based Method

Pedigree-based methods rely on genotypic data from parents and offspring (often trios or extended families) to infer crossover events and build recombination maps [25]. They are among the most widely used methods in livestock populations where multi-generational breeding records are available. In cattle, sex-specific recombination maps were generated in Holsteins, and it was discovered that male cattle had 10% longer recombination maps than females, a pattern opposite to that observed in humans [26]. Similarly, recombination rate differences among male mice from high and low recombination strains were found to be associated with the number of double-strand breaks (DSBs) [27]. 

Despite their utility, pedigree-based methods face limitations. They require large family sizes for sufficient resolution, are expensive due to genotyping costs, and are often impractical in populations with incomplete or missing pedigree records, an issue common in African communal herds. In such populations, the lack of structured mating systems complicates the implementation of this method.

### 4.2. Linkage Disequilibrium-Based Method

LD-based methods use non-random associations of alleles at different loci in a population to estimate historical recombination rates. Tools like LDhat and its successors have been employed in large-scale studies, including the 1000 Genomes Project, to infer fine-scale recombination landscapes [28,29]. A major advantage of LD-based approaches is their ability to work with smaller sample sizes, sometimes as few as 10 sequences, while still generating high-resolution recombination maps [30].

Compared to pedigree-based methods, LD-based approaches can uncover older recombination events and are less dependent on structured breeding designs. However, they can be confounded by the population admixture, selection, and genetic drift, all of which are prominent in African cattle populations such as Nguni and Bonsmara. Without appropriate statistical corrections, these factors can bias LD estimates and mislead inferences of recombination.

### 4.3. Gamete-Based Method

Gamete-based approaches involve the direct sequencing of the sperm or oocytes from a single individual to detect recombination events at the single-cell level. These methods offer the highest possible resolution of recombination mapping and have been successfully used in cattle [31]. In their study, gamete-based sequencing revealed higher recombination rates and finer crossover mapping than pedigree-based data.

While powerful, gamete-based approaches remain limited by costs, technical complexity, and the need for specialised platforms for single-cell isolation and sequencing. Furthermore, they are more difficult to implement in field conditions and therefore less accessible for routine use in livestock breeding, particularly in developing countries. Their use in cattle remains largely experimental, though promising for future applications.

Overall, the method of choice for recombination detection in cattle should be informed by the genomic infrastructure, breeding system, and population structure of the study population, as summarised in Table 3.

In South African beef breeds, where the pedigree information is often sparse and populations are highly admixed, LD-based methods and hybrid approaches integrating SNP marker data and pedigree reconstruction offer practical alternatives. Future innovations such as cost-effective gamete sequencing and integrated recombination-aware selection models may provide more precise tools for harnessing recombination in cattle breeding.

## 5. Single Nucleotide Polymorphism (SNP) Markers in Beef Cattle Genomic Studies

The development of molecular genetic technologies has transformed livestock genomics, introducing multiple generations of molecular markers to study the genetic variation, population structure, and economically important traits in cattle [32]. Early markers such as protein variants and DNA repeat sequences (e.g., microsatellites) played a crucial role in identifying genetic and phenotypic diversity; however, these markers were limited in their genome coverage, resolution, and reproducibility [32].

Among the most prominent DNA sequence polymorphisms now used in livestock genomics are single nucleotide polymorphisms (SNPs), which represent single-base-pair changes at specific loci across the genome [33]. Since their introduction in 1996, SNPs have gained wide adoption due to their high abundance, genome-wide distribution, and amenability to high-throughput genotyping [32,33]. Their advantages include an enhanced resolution for genome-wide association studies (GWASs), an improved mapping precision of quantitative trait loci (QTL), and more accurate genomic predictions. Compared to traditional markers such as microsatellites, SNPs are more stable, less prone to scoring errors, and provide a denser genomic coverage. While whole-genome sequencing (WGS) offers a more comprehensive view of genomic variation, it remains cost-intensive and less accessible for routine applications. Hence, SNP panels serve as a practical and effective alternative in genomic studies, especially for large-scale applications and recombination mapping.

Recent advances have led to the development of commercially available SNP arrays (Table 4), ranging from low- to high-density platforms, including the Illumina BovineSNP50 BeadChip and GeneSeek Genomic Profiler (GGP). These tools have enabled high-resolution studies across diverse cattle populations. For instance, Chao et al. [34] employed the GGP 50K BeadChip to verify sire pedigrees in Taiwanese dairy cattle, reducing the pedigree recording error by 27.78%. Such studies underscore the broad utility of SNP panels in both pedigree verification and trait mapping.

Beyond applications in parentage verification and breed composition, SNPs play a central role in recombination studies. Meiotic recombination is essential for generating genetic diversity and ensuring accurate chromosomal segregation during gametogenesis. In cattle, the variation in recombination rates and hotspot localisation is under strong genetic control. Genome-wide association studies have revealed that specific SNPs are associated with recombination phenotypes and, consequently, fertility outcomes. For example, Weng et al. [35] used the Illumina 50K BeadChip in Angus and Limousin cattle to identify SNPs associated with recombination rate variation and genes such as *RAD51C*, *RAD52C*, and *XRCC3*, which are critical for DNA repair during the meiotic crossover.

One of the most prominent findings in this field is the identification of SNPs near *RNF212*, a gene located on bovine chromosome 10 that stabilises crossover sites during meiosis. This locus was first reported by Sandor et al. [2] and was subsequently validated as a major determinant of the recombination rate variation in both sexes [26,37]. Additional key regulators of recombination include *PRDM9*, *REC8*, and *HEI10* genes. For instance, *PRDM9*, a histone methyltransferase that initiates recombination hotspots, has been shown to exert sex-specific effects in cattle [26]. Likewise, *REC8*, a meiosis-specific cohesin, has been associated with chromatid separation and fertility variation [37]. Several SNPs located on the pseudoautosomal region (PAR) and X-specific regions of the bovine X chromosome (BTAX) were significantly associated with sire fertility, highlighting genes such as FAM9B, TBL1X, and PIH1D3, which are involved in testosterone regulation, spermatogenesis, and sperm motility. These findings demonstrate that BTAX plays a crucial role in explaining fertility variation among bulls and that incorporating X-linked SNPs into genomic prediction models enhances the accuracy of the fertility trait estimation. Consequently, the X chromosome should be prioritised in genomic studies aimed at improving the male reproductive performance in cattle [38]. Beyond recombination, fertility-linked SNPs, such as those in CEBPG, KALRN, and AFF1, identify genomic regions where recombination may influence reproductive success, potentially via the crossover positioning that ensures chromosome segregation and embryo viability [39]

The recent work by Shen et al. [40] further identified novel loci near *MSH4* and *MEI1*, which are involved in double-strand break repair and synaptonemal complex formation, respectively. These SNPs were also correlated with conception rates and embryonic viability, underscoring their dual relevance for genetic diversity and reproductive efficiency. Although these insights are largely derived from studies in European taurine breeds, they are increasingly relevant to South African cattle populations. SNP-based genomic studies have been successfully applied to investigate the genetic diversity and population structure of indigenous breeds. Using the 50K SNP BeadChip, Makina et al. [8] profiled genomic diversity in Bonsmara, Nguni, and Afrikaner breeds. Sanarana et al. [15] further emphasised the utility of SNP genotyping in elucidating the breed structure and identifying candidate genes of economic importance.

However, SNP-based approaches face certain limitations. These include an ascertainment bias, where panels designed from limited populations may not capture rare or breed-specific variants, particularly in highly admixed African breeds. This issue can be mitigated through a breed-specific SNP discovery, imputation using reference panels, and integration with WGS or low-coverage sequencing.

In South Africa, breeds like Bonsmara and Nguni, valued for their productivity and adaptability, stand to benefit from incorporating recombination-associated SNPs into genomic prediction models. Identifying and functionally annotating SNPs near key recombination genes (e.g., *PRDM9*, *DMC1*) enables a better understanding of how meiotic processes modulate allelic combinations underlying complex traits such as fertility, growth, and tick resistance.

Ultimately, integrating SNP data into recombination studies provides a robust framework for identifying selection targets and enhancing the design of breeding programmes tailored to South African beef production systems. As SNP technology continues to evolve, its application in dissecting recombination landscapes will remain pivotal for promoting genetic gain, reproductive efficiency, and long-term resilience in both commercial and indigenous cattle populations.

## 6. Kinship Analysis Using SNP Genetic Markers

A kinship analysis based on single nucleotide polymorphism (SNP) markers has emerged as an indispensable genomic tool, particularly in studies exploring meiotic recombination in livestock. Accurate reconstruction of familial relationships—especially parent–offspring trios—is foundational for mapping crossover events and identifying recombination hotspots. These analyses are critical in genetically diverse cattle populations where pedigree records are often incomplete or absent due to historical breeding practices. Without reliable kinship data, the resolution and reliability of recombination mapping are substantially compromised. Therefore, the SNP-based kinship analysis provides a prerequisite framework for examining the heritable landscape of recombination and its influence on productivity traits in these indigenous and composite beef breeds.

The kinship coefficient, also known as the coefficient of consanguinity, refers to the probability that two alleles sampled from two individuals at the same locus are identical by descent [41,42]. This metric has traditionally been applied to detect pedigree inconsistencies, identify complex familial relationships, verify breed purity, and support conservation efforts. With the advent of high-throughput genotyping technologies, kinship estimation has undergone a transformative shift. SNPs offer several advantages over microsatellites, including a greater abundance across the genome, lower mutation rates, and compatibility with automation [33]. These properties have made SNPs the markers of choice for relationship testing and pedigree reconstruction in livestock species.

In cattle, studies have demonstrated that a panel of approximately 100 carefully selected SNPs can provide more than 99% accuracy in parentage verification [36]. However, the efficacy of such panels can vary across breeds due to differences in allele frequencies and patterns of linkage disequilibrium. This is particularly relevant for South African beef cattle, where a substantial admixture and adaptive divergence are observed. Consequently, the development and application of breed-specific or region-specific SNP panels are essential for ensuring high-resolution kinship and recombination studies in Bonsmara and Nguni populations.

As shown in Table 5, several analytical frameworks have been developed for an SNP-based pedigree analysis, including exclusion-based, relatedness-based, and likelihood-based methods [43]. Among these, likelihood-based approaches are considered the most robust, especially when genotypic data for both parents are available. These methods rely on multilocus genotype probabilities to distinguish between multiple candidate parents and are less prone to errors in the presence of missing data or genotyping inconsistencies [44]. Relatedness-based methods, though limited to pairwise comparisons, remain useful in identifying second-degree relationships such as half-siblings, aunts/uncles, and grandparents [45,46,47].

Importantly, the kinship analysis extends beyond pedigree verification to directly support recombination studies. When accurate parent–offspring trios are reconstructed using SNP data, crossover events can be inferred by comparing the offspring’s haplotype to those of the parents. This enables the precise localisation of recombination breakpoints, allowing for the estimation of genome-wide recombination rates and hotspot identification. Such recombination profiling is essential for understanding the underlying architecture of complex traits. For example, recombination plays a crucial role in reshuffling allelic combinations that affect fertility, growth rate, feed efficiency, and disease resistance traits of considerable economic and adaptive importance in beef production systems [52,53].

In South African beef cattle, where the genetic diversity is high and the pedigree documentation may be lacking, the SNP-based kinship analysis offers a powerful solution. It facilitates haplotype phasing and enables researchers to track recombination dynamics with greater accuracy. Such insights are particularly valuable for indigenous and composite breeds like Nguni and Bonsmara, which have evolved under diverse environmental pressures and management systems. By revealing breed-specific recombination patterns and the loci involved, the SNP-informed kinship analysis enhances the design of genomic selection programmes tailored to the needs of local production environments.

Integrating the kinship analysis with meiotic recombination research in South African beef cattle is vital for advancing precision breeding. It bridges gaps in lineage data, refines the estimation of recombination rates, and ultimately contributes to the improvement of traits related to productivity and resilience in these economically significant breeds.

## 7. Proteins Involved in Mammalian Meiotic Recombination

Meiotic recombination is a tightly regulated, protein-dependent process essential for generating genetic diversity and maintaining chromosomal stability during gametogenesis. The process is orchestrated by a suite of proteins involved in the double-strand break (DSB) formation, homologous chromosome recognition and synapsis, strand invasion, and crossover resolution. In bovines, particularly Nguni and Bonsmara cattle, understanding the functional dynamics and polymorphisms of these proteins has significant implications for mapping recombination landscapes and improving genomic selection models in beef breeding programmes.

Recombination is initiated during the leptotene–zygotene transition of meiotic prophase I, when *SPO11* catalyses programmed DSBs. This meiosis-specific topoisomerase-like enzyme cleaves DNA to initiate homologous recombination [54,55]. The *SPO11* function is supported by auxiliary proteins, including MEI4, *REC114*, and the MRE11–RAD50–NBS1 (MRN) complex, which facilitate DSB formation and processing. In cattle, *SPO11* expression has been confirmed in the testicular tissue, and its activity is fundamental to crossover formation, a key determinant of the recombination rate and distribution [26].

Following the DSB formation, the homologous strand invasion and repair are mediated by recombinases *RAD51* and *DMC1*. These proteins promote interhomolog interactions by facilitating the homology search and strand exchange, ensuring that DSBs are resolved using homologous chromosomes rather than sister chromatids [24]. In bovine studies, orthologues of *DMC1* and *RAD51* have been identified and are expressed in germline tissues. Moreover, polymorphisms in these genes—such as in *RAD51C*, a paralogue involved in the DNA repair pathway choice—have been associated with inter-individual variability in the recombination frequency in cattle [35].

**Figure 4:**
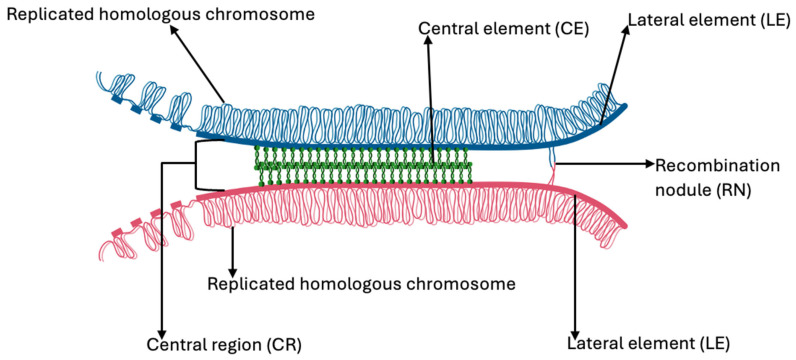
A representation of the synaptonemal complex.

The alignment and synapsis of homologous chromosomes are regulated by the synaptonemal complex (SC), a tripartite proteinaceous structure consisting of two lateral elements (LEs) and a central region (CR). The LEs are composed of proteins *SYCP2* and *SYCP3*, while the CR includes *SYCP1*, *SYCE13*, and *TEX11*, all of which coordinate chromosome pairing and synapsis [56,57] (Figure 4).

In mammals, mutations in *SYCP3* lead to meiotic arrest and infertility. In cattle, *SYCP3* is expressed in the testes, and its structural integrity is necessary for the correct alignment of homologous chromosomes, making it a critical component of the meiotic recombination machinery [56]. Another pivotal protein is *PRDM9*, a histone methyltransferase that directs the localisation of recombination hotspots through the epigenetic marking of DNA at specific binding motifs. *PRDM9* is polymorphic in cattle, and the variation in its zinc finger domain has been associated with sex-specific differences in the hotspot usage and recombination rate across the genome [26]. Additionally, *XRCC3*, a *RAD51* paralogue involved in Holliday junction resolution, has been linked to crossover frequency modulation in bovines, making it a candidate gene for influencing recombination outcomes in breeding contexts [35].

Table 6 summarises key recombination proteins, their functions, and known roles in cattle. These proteins are not only conserved at the sequence level but also exhibit functional relevance in the bovine genome, as evidenced by SNP-based studies and expression profiling in reproductive tissues. 

Understanding the variation in the expression and functionality of these meiotic proteins is particularly important in Nguni and Bonsmara cattle, which are valued for their adaptability, reproductive efficiency, and genetic heterogeneity. Differences in the recombination rate and hotspot localisation shaped by polymorphisms in genes such as *PRDM9*, *RAD51C*, and *SYCP3* may underlie the phenotypic variation in fertility, heterosis, and resilience traits. By incorporating these genetic markers into genomic prediction models, breeding programmes can harness recombination-mediated genetic gain more effectively.

Thus, meiotic recombination proteins serve not only as molecular mediators of chromosomal exchange but also as genomic targets for improving the selection accuracy and genetic progress in beef cattle populations.

## 8. Recombination Features in Beef Cattle

The study of meiotic recombination in beef cattle has gained prominence due to its critical role in shaping genetic diversity, influencing trait heritability, and enhancing breeding efficiency. Recombination affects the segregation of alleles across generations, thereby modulating genetic gain, particularly for polygenic traits. Understanding the underlying genetic architecture of recombination is thus fundamental for the optimisation of genomic selection programmes.

Genome-wide association studies (GWASs) and high-density single nucleotide polymorphism (SNP) arrays have been the primary tools for investigating the recombination rate variation, crossover distribution, and hotspot usage in cattle populations. Early landmark studies, such as that by Sandor et al. [2], used 19,487 SNPs to evaluate recombination landscapes in 10,192 Dutch and 3783 New Zealand bulls, while Weng et al. [35] employed the Illumina BovineSNP50 BeadChip in 2778 Angus and 1485 Limousin sire–offspring pairs to identify recombination-associated genes, including *SPO11*, *RNF212*, *XRCC3*, *RAD51C*, and *PRMT8*. These studies revealed that recombination is influenced by multiple loci, with evidence of breed-specific genetic effects.

One of the most extensively studied recombination regulators is *PRDM9*, which governs the hotspot placement via its zinc finger domain. In cattle, four *PRDM9* paralogues have been identified, with the paralogue on chromosome 1 showing signs of positive selection [60]. Its role was further confirmed in Holstein populations, where *PRDM9* influenced the crossover localisation and frequency. Additional loci such as *RNF212B*, a paralogue of *RNF212*, have also been identified as recombination modulators [37].

Kadri et al. [37] provided insight into recombination rate differences between sexes and species. In cattle, males exhibit longer genetic maps (~23.3 Morgans) compared to females (~21.4 Morgans), a trend also observed across livestock species. In sheep, average genome-wide recombination rates are approximately 1.6 cM/Mb, whereas pigs show slightly lower rates around 1.1–1.3 cM/Mb [37,61]. These differences are partly attributed to species-specific chromosomal architectures and the recombination suppression near centromeres.

To quantify recombination events, several statistical approaches are utilised. Pedigree-based methods estimate recombination rates using inherited SNPs across generations, while linkage disequilibrium (LD)-based methods, such as LDhat and LDhelmet, infer historical recombination patterns from population-level genotype data. More recently, haplotype-based hidden Markov models (HMMs), such as those implemented in fastPHASE, and Bayesian inference tools, including BEAGLE, have been applied to infer recombination events with a high precision.

Beyond SNP-based genotyping, novel genomic and epigenomic technologies are emerging as powerful tools to elucidate the molecular regulation of recombination. Transcriptomic profiling (RNA-seq) can reveal the temporal expression dynamics of recombination-associated genes such as *DMC1*, *HFM1*, and *MLH3* during meiotic prophase I, particularly in testicular tissue. This approach enables the identification of the breed-specific expression QTLs that may influence recombination outcomes.

Similarly, DNA methylation and chromatin accessibility assays (e.g., bisulfite sequencing, ATAC-seq) offer insights into the epigenetic regulation of the hotspot activity. In marine, mice, and human studies, the methylation at *PRDM9* binding motifs has been shown to suppress the hotspot activity [62], and similar mechanisms are likely to operate in cattle, though these remain underexplored. The integration of multi-omics data, such as methylome and transcriptome profiles with SNP genotyping, holds promise for a systems-level understanding of recombination biology in livestock.

Despite these advancements, the recombination research in African beef cattle remains underdeveloped. Indigenous breeds such as Bonsmara and Nguni exhibit unique genomic backgrounds shaped by historical admixture, natural selection, and environmental adaptation. To date, no recombination-focused GWAS has been conducted in these populations, leaving a gap in understanding whether recombination loci identified in Holstein or Angus cattle are conserved or breed-specific (Table 7).

Future research in South Africa should employ high-density SNP arrays and low-coverage whole-genome sequencing to characterise the recombination variation in structured Bonsmara and Nguni populations. Analyses should incorporate both a pedigree-based recombination rate estimation and LD-based haplotype reconstruction, with a focus on candidate genes such as *PRDM9*, *RNF212*, and *REC114*. Coupling SNP data with transcriptomic and epigenomic profiling during gametogenesis will be instrumental in identifying regulatory variants affecting recombination.

Such integrative genomic studies are crucial for informing recombination-aware genomic selection and for designing breeding strategies that account for the recombination variability in locally adapted cattle. Ultimately, this will enhance the sustainability and resilience of beef production systems across Southern Africa.

## 9. Practical Implications of Understanding Regulatory Mechanisms of Meiotic Recombination in Cattle

Understanding the regulatory architecture of meiotic recombination in cattle has far-reaching implications for genetic improvement, genomic selection, and the long-term sustainability of breeding programmes. Recombination plays a central role in reshuffling alleles during meiosis, generating novel haplotypes and thus enhancing genetic diversity—an essential resource for responding to changing environmental conditions and evolving disease pressures [62]. As such, elucidating the mechanisms that govern the recombination rate, crossover distribution, and hotspot activity provides actionable insights for optimising selection schemes and managing the linkage disequilibrium (LD) in breeding populations.

One of the most immediate applications of recombination research lies in improving the accuracy and efficiency of genomic selection. Current prediction models often assume uniform recombination rates across the genome, yet recombination is highly heterogeneous, with hotspots that can influence marker–trait associations and the mapping resolution [2,37]. By integrating empirical recombination data into genomic prediction pipelines, particularly those derived from high-resolution maps or inferred from large SNP datasets, it becomes possible to enhance the imputation accuracy, reduce the bias in marker effect estimations, and increase the reliability of genomic estimated breeding values (GEBVs) [27,63].

Additionally, the knowledge of recombination regulation can inform strategies to mitigate inbreeding depression. In small or closed cattle populations, regions of suppressed recombination, often due to chromosomal rearrangements or *PRDM9*-associated hotspot clustering, can lead to haplotype fixation and the loss of genetic variability. Targeted selection for alleles that promote higher genome-wide recombination, such as favourable haplotypes at the *RNF212* or *PRDM9* loci, may counteract this effect by increasing effective recombination and thereby maintaining heterozygosity in critical genomic regions [64,65].

Moreover, from a practical breeding perspective, manipulating the recombination rate holds promise for decoupling unfavourable genetic linkages. For example, traits of economic relevance, such as the growth rate and fertility, are sometimes antagonistically linked due to tight genetic linkage or pleiotropy. Recombinogenic genotypes may enhance the likelihood of breaking such linkages, facilitating the selection of animals that exhibit optimal trait combinations without compromising other important attributes.

Emerging gene editing technologies further suggest a potential for the direct manipulation of recombination-related loci. Although still largely theoretical in livestock, the CRISPR/Cas9-mediated modification of recombination regulators, as demonstrated in murine and plant models, opens the possibility for engineering recombination landscapes to favour desirable haplotype configurations in cattle. However, ethical, regulatory, and biological considerations must be carefully addressed before such approaches can be deployed at scale.

Finally, recognising sex-specific recombination dynamics enables more nuanced reproductive strategies. Since recombination rates and patterns differ markedly between bulls and cows, often with males exhibiting longer genetic maps, breeding programmes could exploit this dimorphism by selecting sires with optimal recombination profiles, thereby increasing genetic gain per generation. The practical utility of understanding meiotic recombination in cattle lies not only in improving the fundamental knowledge of genome biology but also in its translational potential to optimise breeding outcomes, enhance resilience, and ensure the sustainability of livestock production systems in the face of global challenges.

## 10. Conclusions and Recommendations

This review highlights that the current knowledge on meiotic recombination in South African beef cattle, specifically Bonsmara and Nguni, is insufficient, despite its critical role in promoting genetic diversity, enhancing trait heritability, and supporting environmental adaptability. While global studies have identified major recombination-associated genes, such as *PRDM9*, *RNF212*, and *RAD51C*, there is a lack of breed-specific data for African cattle, limiting the application of genomic selection in these populations.

To bridge this gap, future research should prioritise the development of high-resolution recombination maps using SNP-based genotyping, the functional characterisation of key loci, and the integration of findings into genomic prediction models. Incorporating reproductive biotechnologies, such as in vitro fertilisation and cloning, will further support mechanistic studies of recombination. These efforts hold significant potential to improve precision breeding, drive sustainable productivity, and align genomic innovations with the resilience needs of the South African beef industry.

## Figures and Tables

**Table 1:** The total number of Bonsmara cattle in South Africa over the years, adopted from (https://dad.fao.org/, accessed on: 16 February 2024).

Year	Population		Sex		Registered Pure Bred Females
	Minimum	Maximum	Male	Female	
2018	52,000	85,000	10,000	42,000	42,000
2021	136,481	183,500	36,359	100,122	100,122
2022	1000	136,348	36,660	98,992	98,992
2023	10,000	142,849	39,707	103,142	103,142

**Table 2:** Differences between the Bonsmara and Nguni breeds relevant to meiotic recombination.

Characteristic	Bonsmara	Nguni
Breed Type	Synthetic composite (Afrikaner × Shorthorn × Hereford)	Indigenous (Sanga type; Bos taurus × Bos indicus)
Origin	Developed in South Africa through structured breeding	Ancient breed introduced via historical migration
Genetic Diversity	Moderate heterozygosity; well-characterised by SNP panels	High genetic diversity; low inbreeding levels
Adaptability	High adaptability to subtropical climates	Exceptional tolerance to harsh climates and poor nutrition
Production System	Predominantly commercial and intensive systems	Mixed systems: smallholder, communal, and semi-commercial
Body Size (Mature Males)	Large (≈950 kg)	Medium (≈500–700 kg)
Reproductive Traits	Early sexual maturity; strong maternal traits	High fertility; low calf mortality at first calving
Disease Resistance	Tick and tick-borne disease tolerance	Natural tick resistance and general disease resilience
Phenotypic Traits	Uniform red/brown coat; good beef conformation	Diverse coat colours and patterns; hardy morphology
Genomic Resources	Extensively studied with SNP-based tools	SNP resources available; limited fine-scale recombination data
Relevance to Recombination Studies	Ideal for mapping recombination and trait associations	Valuable for understanding polygenic adaptation via recombination

**Table 3:** Comparison of methods for detecting recombination in livestock genomics.

Method	Input Data	Resolution	Cost	Strengths	Limitations	Applicability in African Cattle
Pedigree-Based	Parent–offspring genotypes	Moderate to High	High	Accurate; captures sex-specific recombination	Requires large pedigrees, costly genotyping, limited resolution with small families	Low (due to incomplete pedigrees)
LD-Based	Population-level SNP genotype data	High (historical)	Moderate	Small sample size sufficient; fine-scale mapping	Sensitive to admixture, selection, and population structure	Moderate (requires population-specific calibration)
Gamete-Based	Sequenced sperm/ova and donor genome	Very High	Very High	Direct detection of crossovers; no pedigree needed	Technically demanding; expensive; low throughput	Low (experimental use; promising future application)

**Table 4:** Commonly used SNP panels in cattle genomics and their applications.

SNP Panel	No. of SNPs	Manufacturer	Applications	References
Illumina BovineSNP50 BeadChip	~54,000	Illumina	Parentage verification, breed composition, genomic diversity, QTL mapping, recombination rate estimation	[8,35]
GeneSeek Genomic Profiler (GGP) 50K	~50,000	Neogen/GeneSeek	Pedigree accuracy, trait mapping, genomic selection	[34]
Illumina BovineHD BeadChip	~777,000	Illumina	High-resolution mapping, recombination hotspot identification, fine-scale linkage disequilibrium and haplotype analysis	[26,36]
Custom SA Breed-Specific SNP Arrays	Varies	Academic/Private	Genomic characterisation of indigenous breeds, validation of local trait-associated SNPs, recombination trait mapping in Nguni and Bonsmara breeds	[15]

**Table 5:** A brief outline of global studies that employed SNP markers for a kinship analysis in livestock species, highlighting their application in the pedigree verification, parentage assignment, and estimation of breed composition.

Species	Title of the Study	Reference
Swine	Kinship coefficient of Landlly pigs using porcine 60K SNP Beadchip and pedigree data.	[48]
Cattle	Pedigree verification and parentage assignment using genomic information in the Mexican Holstein population.	[36]
Cattle	SNP panels for the estimation of dairy breed proportion and parentage assignment in African crossbred dairy cattle.	[49]
Cattle	Use of SNP genotyping to determine pedigree and breed composition of dairy cattle in Kenya.	[50]
Cattle	Sire pedigree error estimation and sire verification of the Taiwan dairy cattle population by using SNP markers.	[34]
Cattle	Testing different single nucleotide polymorphism strategies for prediction of genomic breeding values in dairy cattle based on low-density panels.	[51]

**Table 6:** Summary of key meiotic recombination proteins and their functions in mammals and cattle.

Protein	Function	Known in Cattle?	Phenotype When Mutated (in Mammals)	References
*SPO11*	Initiates programmed DSBs	Yes	Meiotic failure, infertility	[54,55]
*DMC1*	Facilitates homologous strand invasion	Yes	Arrest in meiotic prophase I	[24]
*RAD51*	DSB repair; promotes strand exchange	Yes	Chromosome mis-segregation	[20]
*SYCP3*	Lateral element of SC	Yes	Aneuploidy, infertility	[56,58]
*SYCP1*	Central element of SC	Yes	Failed synapsis	[57]
*TEX11*	Synapsis and crossover regulation	Likely	Azoospermia in humans	[59]
*PRDM9*	Hotspot localisation	Yes (polymorphic)	Altered recombination landscape	[26]
*XRCC3*	Holliday junction resolution	Yes	Recombination deficiency	[35]
*RAD51C*	Promotes DSB repair pathway choice	Yes	Reduced CO formation	[35]

**Table 7:** Candidate genes associated with meiotic recombination in global cattle breeds.

Gene	Function	Breed(s)	Sex-Specific?	References
*PRDM9*	Hotspot placement and regulation	Holstein, Angus	Both	[35,37]
*RNF212*	Crossover designation	Limousin, Holstein	Male-biassed	[35]
*RNF212B*	Paralogue of *RNF212*	Holstein	Male	[37]
*RAD51C*	DSB repair and homologous recombination	Angus, Limousin	Both	[35]
*REC114*	DSB initiation scaffold	Holstein	Both	[35]
*GCLM*	Glutathione biosynthesis, possibly regulating DSBs	Holstein	Both	[35]
*HFM1*	Crossover maturation	Holstein	Male	[37]
*MLH3*	Crossover resolution	Holstein	Male	[37]
*CPLX1*	Synaptic vesicle regulation	Holstein	Both	[35]
*MSH4/MSH5*	Crossover stabilisation and mismatch repair	Holstein	Sex-specific	[37]

## Data Availability

No new data was generated for this review.
